# Different mulch films, consistent results: soil fauna responses to microplastic

**DOI:** 10.1007/s10661-024-13096-x

**Published:** 2024-09-18

**Authors:** Antonia Weltmeyer, Martina Roß-Nickoll

**Affiliations:** Institute for Environmental Research, RWTH Aachen University, Aachen, Germany

**Keywords:** Mulch film microplastic, Biodegradable plastic, Earthworm, Collembola, Reproductive toxicity, Microplastic in soil

## Abstract

Agricultural activities contribute to plastic pollution, with unintentional introduction and intentional use of plastic mulch films leading to the accumulation of microplastic particles in soils. The lack of removal techniques and scarce information on the effects on soil organisms, especially for biodegradable mulch films, necessitate an assessment of potential effects. This study aimed to elucidate the effects of mulch film microplastic on soil fauna by investigating reproduction output and subcellular responses before and after recovery from exposure. Two common soil organisms, *Folsomia candida* and *Eisenia fetida*, were exposed to petroleum-based polyethylene (PE) and biodegradable polylactic acid/polybutylene adipate terephthalate (PLA/PBAT) microplastic for 28 days, according to OECD guidelines 232 and 222, respectively. Juvenile numbers revealed no polymer- or concentration-dependent effects on *E. fetida* and *F. candida* reproduction after exposure to up to 5 and 10 g/kg_dw_ soil, respectively. To provide a more sensitive and early indication of sublethal effects, subcellular responses in *E. fetida* were analyzed. Glutathione S-transferase (GST) activity increased with rising microplastic concentration; however, catalase (CAT), acetylcholine esterase (AChE) activity, and reactive oxygen species (ROS) did not differ from control levels. Further, the more environmentally relevant PE polymer was chosen for in-depth assessment of subcellular response after 28-day microplastic exposure and subsequent 28 days in uncontaminated soil with *E. fetida*. No significant differences in biomarker activity and stress levels were observed. We conclude that mulch film–derived microplastic did not adversely affect earthworm and collembolan species in this scenario, except for a slight induction in the detoxification enzyme glutathione S-transferase.

**Supplementary Information:**

The online version contains supplementary material available at 10.1007/s10661-024-13096-x.

## Introduction

Although large-scale plastic production started only about 70 years ago, a world without plastic is now unimaginable. Following today’s trend, in 2050, Geyer et al. (2017) estimate that 26,000 Mt of primary plastic waste will have been generated and 12,000 Mt discarded in landfills and in nature itself. In 2022, agriculture, farming, and gardening were responsible for about 3.1% of all used plastics in Europe (Plastic Europe, 2022). Although not representing a considerable share, these plastics are in direct contact with soils and water, posing an accumulation potential within these systems. Moreover, Horton et al. (2017) estimated that the share of released plastics is 4 to 23 times higher in terrestrial ecosystems compared to oceans.

Diverse sources of plastic input exist on agricultural soils, including unintentional introductions by wind drift or sewage sludge application and intentional introductions through garden foils and mulch films.

Mulch films are thin—usually between 12 and 80 µm thick—plastic films that are mainly used to improve water efficiency, regulate soil temperature, and suppress weed growth (Espi et al., 2016). Due to their significant advantages in challenging climates, they have become an indispensable agricultural practice over the years (Huerta Lwanga et al., 2022). The market began to grow in the early 1950s with the commercialization of low-density PE and its utilization for mulching vegetables. Nowadays, the agricultural film market is still expected to grow by 7.4% yearly from 2022 to 2031 (Transparency Market Research, 2022). However, due to their low thickness and regular application with partly incomplete removal, accumulation of smaller parts and, finally, microplastic particles (MPP) in agricultural soils occurs. A modeling study by Brandes et al. (2021) estimated that about 5–9 mg/kg of plastic debris from agricultural plastic covers are added to German agricultural soil every year.

A way to target the problem of plastic accumulation is the usage of biodegradable polymers. The main advantages of these materials are their limited lifetime and subsequent possible transformation to H_2_O, CO_2_, and methane. The production of biodegradable polymers increased, after stagnation due to the COVID-19 pandemic, from 0.8 (2018) to 1.3 (2021) million tons (Plastic Europe, 2022). According to Skoczinski et al. (2023), the most promising biobased biodegradable polymer is polylactic acid (PLA), commonly applied in plastic biodegradable mulches (BDM). PLA can be both biodegradable and biobased and is commercially successful due to its good processability and mechanical properties (Jem & Tan, 2020).

The agronomic performance of BDM is comparable to PE films (Moreno & Moreno, 2008; Saglam et al., 2017), thus offering a sustainable alternative due to their inherent degradation properties and prevention of agricultural plastic pollution. A recent focus has been the realistic degradation of BDM in the field since incomplete degradation would lead to (micro)plastic accumulation in soil, which can hardly be removed from soil (de Souza Machado et al., 2018).

Microplastics in soil can have various effects on the soil environment. On the microbial scale, it can shift the community structure, increase microbial biomass, and decrease microbial and bacterial biodiversity. As for soil fauna, effects are almost exclusively negative, such as a decrease in growth or reproduction as summarized by Liu et al. (2023).

Observable effects are highly dependent on polymer type, shape, and size, complicating direct comparisons (Ji et al., 2021). Mulch film is conventionally based on PE, which has been shown to cause various effects on common soil species, such as earthworms and springtails (Yang et al., 2023; Cheng et al., 2020; Ju et al., 2019; Kim & An, 2019 and 2020). The studies’ endpoints differ between species, but a decrease in reproduction output was observed for both species as it is one of the most investigated endpoints in soil organisms (Liu et al., 2023).

Biodegradable and biobased MPP have only been investigated recently. Earthworm studies have only started to grow in the last years (Ding et al., 2021, Liwarska-Bizukojc et al., 2023, Holzinger et al., 2023), and are still scarcely found for springtails (Schnepf et al., 2022) or other soil organisms.

Further, most studies investigate purchased pristine MPP, thereby creating more artificial scenarios and decreasing the ecological relevance. Exposure to mulch film microplastic has not been published for springtails except for one very recent study by van Loon et al. (2024). For earthworms, most studies with mulch films focused on PE mulch film interaction with pesticides (Cheng et al., 2020; Song et al., 2023). To our current knowledge, only three studies included effects from both conventional and biodegradable mulch films (Forsell et al., 2024; Holzinger et al., 2023; Yu et al., 2022).

Thus, there is a growing need for a deeper understanding of the ecological impacts of microplastics that result from both conventional and biodegradable mulching films.

This study focusses on three objectives. Our first objective is to assess and compare the effects of conventional and biodegradable mulch films on representative soil species. For the comparison of mulch films, two of the most typically used soil test organisms, namely *Eisenia fetida* and *Folsomia candida*, were chosen. Commonly used PE and biodegradable mulch films were selected to represent different application scenarios. Due to the low environmental relevance of acute tests in soil ecosystems, we focused on investigating chronic effects such as changes in weight and reproductive output. Thus, we exposed both species with the frames of OECD guidelines 222 and 232 for 28 days to size-specified MPP from commercially available mulch film.

As a second objective, we want to investigate the sublethal effects of microplastic exposure with *E. fetida*. While reproduction measurements give valuable insights into the species fitness, they cannot detect early biochemical or physiological changes in species, such as subcellular analyses are able to. Studies addressing microplastic effects have shown significant responses to oxidative damage, enzymatic biomarker activity, and neurotoxicity in earthworms (Chen et al., 2020; Cui et al., 2022; Forsell et al., 2024; Rodriguez-Seijo et al., 2018a, 2018b; Yu et al., 2022). For this study, subcellular mechanistic responses of glutathione S-transferase, acetylcholinesterase, catalase, and oxidative stress were carried out. The chosen subcellular analyses have been conducted regularly and proved to be impacted by microplastic exposure (Chen et al., 2020; Lackmann et al., 2022; Yu et al., 2022).

Glutathione S-transferase is an indicator for the detoxification system, acetylcholinesterase inhibition signifies neurotoxicity, catalase activity serves as an indicator of oxidative stress response, and reactive oxygen species informs on cellular damage and oxidative stress mechanisms. Due to their size limitation, it is challenging to assess biomarker expression in springtails, which is why this was only conducted for *E. fetida*.

Our third objective was to assess if earthworms’ subcellular responses can recover from exposure to microplastic. For this matter, the previously exposed earthworms were split into two groups of which one was analyzed on the sport and the other was recovered in uncontaminated soil for another 28 days before analyses of subcellular biomarker expression. To our knowledge, the recovery of soil inhabitants exposed to agricultural MPP has not yet been investigated.

Our results will help elucidate the potential impacts of environmentally relevant plastic applications and provide insight into the possible risks linked to soil microplastic pollution.

## Material and methods

### Microplastic

Microplastic was created by cryomilling two commercially available mulch films based on polyethylene and a polylactic acid/polybutylene adipate terephthalate blend. The size range of the resulting particles was determined with the confocal Raman microscope alpha300 R (WITec GmbH, Germany) at 5 × magnification (Zeiss EC Epiplan- 5 × /0.13). Image analysis was conducted with the Software WITec ParticleScout. Image size was 35.8 × 26.3 mm with 30 × 22 images being stitched, resulting in 660 images, of which 8 were stacked in 500 µm on the *z*-axis for one picture. For the PE microplastic, 403 particles were identified via contrast analysis, of which 270 overlying particles were excluded to reduce adulteration of results due to electrostatic interaction. For the PLA/PBAT microplastic, 448 particles were identified and 138 were deleted, resulting in 310 particles for the size analysis.

Detailed particle size distribution is presented in the Appendix (Tab. A2, Tab. A3). The particles were sieved to a size range of 50 µm to 1 mm with metal sieves and stored in the dark until use. The resulting size distributions are presented in Table 1.

**Table 1:** Percentual size distribution of polyethylene (PE) and polylactic acid/polybutylene adipate terephthalate (PLA/PBAT) particles, which were applied in the biotests

Minimal size of MP (μm)	% total particles	
	PE	PLA/PBAT
50-100	3.37	8.06
100-150	7.87	7.33
150-200	4.49	10.26
200-400	29.21	35.16
400-600	21.35	15.38
600-1000	33.71	23.81

### Test substrate

The chosen test substrate is the RefeSol 01-A (sieved ≤ 2 mm), a slightly loamy sand, medium acid, very slightly humic soil, with physicochemical properties given in Appendix Table 2. It was obtained from Fraunhofer Institute for Molecular Biology and Applied Ecology IME (Schmallenberg, GER). RefeSol soils match the prerequisites in various terrestrial ecotoxicological guidelines. They are applicable for all investigation methods according to BbodSchV and also declared reference soils by the German Federal Environment Agency (Umweltbundesamt, UBA).

### Test organisms

Both *E. fetida* and *F. candida* were chosen as test organisms because of their widespread use in ecotoxicological testing, their ready availability and short reproduction circles. Earthworms were obtained from Wurmwelten.de (Stadtoldendorf, GER). They were bred in commercially available potting soil and fed with cooked organic potato buried in different places of the substrate.

Springtails were obtained from an in-house synchronized breeding facility. They were bred on plasterboard in the dark at 20°C and fed with dry yeast.

### Reproduction test with F. *candida*

The reproduction assay with *F. candida* was conducted according to OECD 232 (2016b) under the described conditions (light/dark cycle 16h:8h and temperature 20 °C). The microplastic was added and mixed with the dry soil at final concentrations of 0.1, 1, 5, and 10 g MP/kg soil_dw_ and the soil was then replenished with deionized water up to 50% of the water holding capacity (ca. 145 g H_2_O/kg soil_dw_). A particle control with kaolin and sand particles at 10 g/kg soil_dw_ was conducted additionally within one test run as well as one test with a concentration of 0.02 g MP/kg soil_dw_. In short, 10 synchronized juveniles (9–12 days old) per technical replicate were exposed for 28 days with 30 g exposure soil_dw_. They were fed with a spatula tip of dry yeast each week and aerated trice weekly. Test vials, which fell over while open during feeding and watering were excluded from analysis.

All concentrations were tested on 3 test days with 4 technical replicates, except the negative control with 8 technical replicates.

### Reproduction test with E. fetida

The reproduction assay with *E. fetida* was conducted for 28 days with 10 adult animals per vial, according to OECD 222 (2016a) and under the described conditions (light/dark cycle 16h:8h and temperature 20 °C). The microplastic was added and mixed with the dry soil at final concentrations of 0.5, 1, 2, and 5 g MP/kg soil_dw_ and the soil was then replenished with deionized water up to 50% of the water holding capacity. In short, 10 adult worms in each of the 3 technical replicates per concentration were exposed for 28 days with 500 g exposure soil_dw_. Negative controls were exposed to uncontaminated soil with 10 worms in each of the 3 technical replicates. For PE and PLA/PBAT microplastic exposure, tests were conducted on 2 and 3 test days, respectively, resulting in 6 and 9 replicates per concentration.

Earthworms were fed with 5 g of cooked organic potato each week, which was incorporated into the upper soil layer.

The soil was sieved with a 2 mm and 1 mm mesh size and washed out and cocoons were collected after exposure. Cocoons were kept on moist filter paper and checked at least weekly for hatchlings. The data were collected to investigate the influence of microplastic concentration and type on hatching date.

### Biomarker analysis

#### Chemicals

The following chemicals were used for the biomarker and oxidative stress analysis:

Sodium phosphate monobasic dihydrate (NaH_2_PO_4_·2H_2_O, CAS 13472–35-0), disodium hydrogen phosphate (NaH_2_PO_4_, CAS 7558–79-4), 5,5′-dithiobis-(2-nitrobenzoic acid) (DTNB) ([-SC_6_H_3_(NO_2_)CO_2_H]_2_, CAS 69–78-3), (2-mercaptoethyl)trimethylammonium iodide acetate (acetylthiocholine iodide) (CH_3_COSCH_2_CH_2_N(CH_3_)_3_I, CAS 1866–15-5), 1-chloro-2,4-dinitrobenzene (CDNB) (C_6_H_3_ClN_2_O_4_, CAS 97–00-7), (2S)-2-amino-4-{[(1R)-1-[(carboxymethyl)carbamoyl]-2-sulfanylethyl]carbamoyl}butanoic acid (glutathione (GSH)) (C_10_H_17_N_3_O_6_S, CAS 70–18-8), sodium dihydrogen phosphate dihydrate (NaH_2_PO_4_ × 2H_2_O, CAS 13472–35-0), hydrogen peroxide (H_2_O_2_, CAS 7722–84-1), Invitrogen™ CM-H_2_DCFDA (C_27_H_19_Cl_3_O_8_, CAS 1219794–09-8) (Thermo Fisher Scientific).

Pierce™ BCA Protein Assay Kit for protein concentration measurements.

#### Sample preparation

An additional exposure recovery scenario was conducted, with worms exposed for 28 days to PE-MP-contaminated soil (0.5, 1, 2, 5 g MP/kg soil) and worms exposed as described and set back in uncontaminated soil for 28 days at the same conditions as previously. This scenario was conducted to investigate the recovery from potential stress through microplastic exposure.

All concentrations tested on different test days with 3 technical replicates to determine repeatability.

For the biomarker analysis, 5 worms per technical replicate (15 worms per concentration/test day) from the recovery exposure scenario were depurated for 24 h and visually examined for the empty gastrointestinal tract. Worms were weighted and homogenized with an Ultra-Turrax T18 homogenizer in cold sodium phosphate buffer (0.1 M, pH 7.2, in ratio 1:4 w:v). Homogenates were subsequently centrifuged (30 min, 4 °C, 4000 g) and the supernatant (post-mitochondrial fraction, S9) was thrice aliquoted and transferred into fresh tubes, which were stored at − 60 °C or above liquid nitrogen until biomarker and oxidative stress analysis.

The following procedures were obtained from Lackmann et al. (2021) and are shortly presented.

#### Glutathion S-transferase (GST)

For the GST activity (Habig & Jakoby, 1981) measurement, 7.5 µL of defrosted sample, 160 µL CDNB (1 mM in sodium phosphate buffer), and 40 µL GSH (25 mM in distilled H_2_O) were combined in triplicates in a 96-well microplate. Directly after application, the absorbance was measured kinetically at room temperature and 340 nm for 90 s (every 15 s). A blank without sample was applied in triplicates per plate. With the corresponding amount of protein, the specific enzyme activity was calculated in nmol of conjugated GSH per minute and mg of protein.

#### Acetylcholine esterase (AChE)

Acetylcholine esterase activity (Ellmann et al., 1961) was determined by the subsequent addition of 7.5 µL defrosted S9 sample, 200 µL sodium phosphate buffer (0.1 M, pH 7.2), 20 µL acetylthiocholine iodide (156 mM), and 10 µL DTNB (1.6 mM) in a 96-well microplate. Directly after combination, the kinetics were determined through absorbance measurements at 412 nm for 1.5 min in 15 s intervals at 20 °C with a *Cytation* 5 Multi-Mode *Reader* (Biotek, Friedrichshall, Germany). The specific AChE activity was calculated using the protein amount of the respective sample and was given nmol of acetylthiocholine iodide hydrolyzed in 1 min per mg of proteins.

#### Catalase (CAT)

For the CAT activity measurements (Claiborne, 1985), 3 µL defrosted sample, 100µL of sodium phosphate buffer (0.1 M, pH 7.2), and 100µL of H_2_O_2_ were applied in triplicates to a UV 96-well microplate. Absorption at room temperature was measured kinetically at 240 nm for 90 s (every 15 s). A blank without sample was applied in triplicates per plate.

With the corresponding amount of protein, the specific enzyme activity was calculated in nmol of degraded H_2_O_2_ per minute and mg of protein.

#### Oxidative stress

For ROS detection, 5 µl defrosted sample, 95 µl sodium phosphate buffer (0.1 M, pH 7.2), and 5µL CM-H_2_DCFDA (7.87 µM) were added to a 96-well microplate in triplicates. Triplicate blanks were performed on each plate. Plates were incubated for 30 min at 25 °C and fluorescence was subsequently measured at 485 nm (excitation) and 530 nm (emission) with a *Cytation* 5 Multi-Mode *Reader.*

#### Protein content

For the protein content determination, the bicinchoninic assay was applied to assess the protein content of each sample in triplicate measurements in 96-well plates. A calibration curve with bovine albumin serum was applied to the first row of each plate. The remaining wells were filled with 1.5 µL of supernatant, 23.5 µL of sodium phosphate buffer (0.1 M, pH 7.2), and 200 µL of the working solution, consisting of BCA reagent A:B (50:1; v:v). After incubation for 120 min at room temperature, absorbance was measured at 562 nm with *Cytation* 5 Multi-Mode *Reader*. Protein contents were calculated using the calibration curve.

#### Statistical analysis

Statistical analysis was performed using GraphPad Prism 9 (GraphPad Inc., San Diego, CA, USA). Total numbers of offspring as well as biomarker response and weight changes were tested for significant differences compared to their respective negative controls using ordinary single factor analysis of variance (ANOVA) and post hoc tests (Tukey’s multiple comparisons test, *p* = 0.05). In the case of heterogeneous variances, a Welch ANOVA with Brown-Forsythe test and a post hoc test with Dunnett’s multiple comparisons were performed. If both the normal distribution and the homogeneous distribution were rejected, the data were analyzed with a Kruskal–Wallis test with Dunnett’s multiple comparisons test. The normal distribution was analyzed with the Shapiro–Wilk test. Outliers were tested with the ROUT method and excluded when confirmed.

## Results

### Influence on the reproduction of springtails and earthworms

**Fig. 1:**
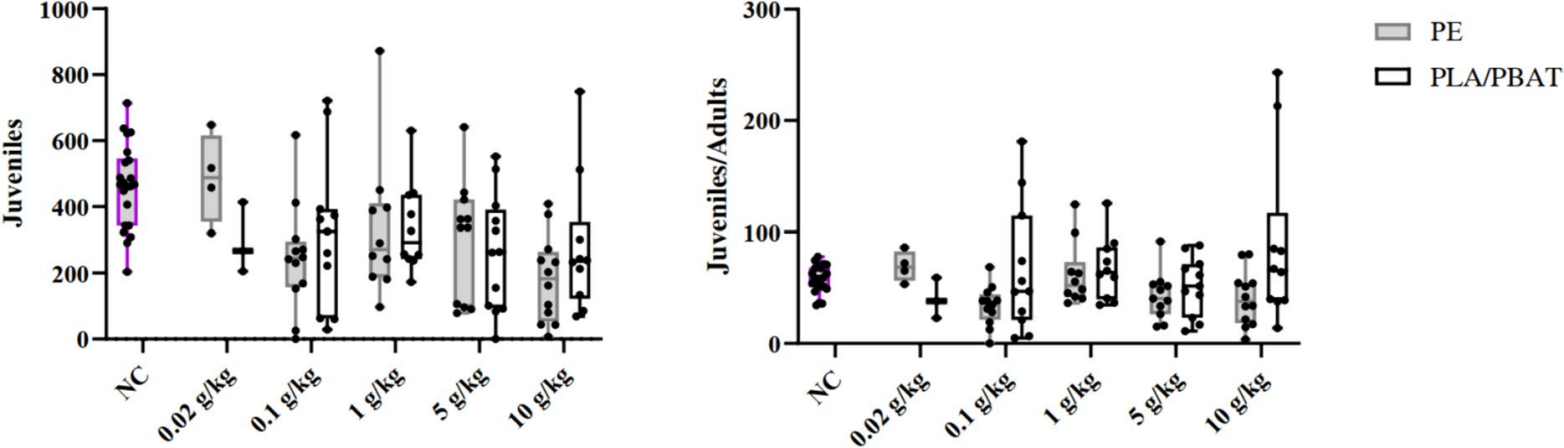
Number of springtail juveniles (left) and juveniles per adult (right) after exposure to either PE (grey) or PLA/PBAT (black) microplastic. Minimum to maximum values are presented with negative controls in purple

For the reproduction tests conducted with springtails, an average adult mortality of 19%, an average juvenile amount of 464 animals, and an average variation coefficient of juveniles of 26% were observed for the negative controls (NC) in the three test days. Therefore, the validity criteria according to OECD (2016b) were fulfilled. Test results are presented in Fig. 1. Both graphs result from the same tests with following concentrations with given replicate number for PE and PLA/PBAT, respectively: negative control (NC, *n* = 22), 0.02 (*n* = 4/*n* = 3), 0.1 (*n* = 12/*n* = 11), 1 (*n* = 10/*n* = 10), 5 (*n* = 11/*n* = 12), and 10 (*n* = 12/*n* = 10) g MP/kg dry soil.

Due to seemingly random variation in the survival of adults within the validity criteria, the chosen way to interpret the data was considered by analyzing the juveniles per adult animal in each test vial.

There were no significant differences between the controls and different PLA/PBAT concentrations. For the PE testing, the concentration of 0.1 g/kg showed significantly lower (*p* = 0.0203) amounts of juveniles per adult compared to the negative control. The pooled replicate samples for 1 g PE/kg and 10 g (PLA/PBAT)/kg were not normally distributed which is why Welch-ANOVA was applied as well, which resulted in the same finding for NC vs. 0.1 g PE/kg (*p* = 0.0136) and no other significant differences.

**Fig. 2:**
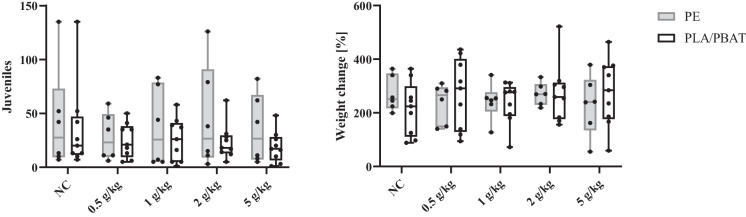
Number of earthworm juveniles (left) and adult weight change (right) after exposure to either PE (grey, *n* = 6) or PLA/PBAT (black, *n* = 9) microplastic in concentrations from 0, 0.5, 1, 2, and 5 g MP/kg dry soil

Results from the reproduction assays with *E. fetida* are presented in Fig. 2 with juvenile number and weight change of adults after 28 days of exposure. Both parameters did not change significantly after exposure to PE or PLA/PBAT microplastic in soil.

Further, there were no differences in the numbers of juveniles per cocoon between the concentrations or the polymer types. The hatching took place from week 2 up until the termination of monitoring at week 20 after cocoon collection (see Appendix Fig. 5). Total amount of hatched juveniles per week could not be linked to exposition scenario or polymer type. Overall, most juveniles hatched after 5 weeks (median: 6.5, mean: 8.6, *n* = 75), being significantly more compared to any other week except for week 4 (median: 4.0, mean: 4.6, *n* = 45).

### Influence on biomarker expression

**Fig. 3:**
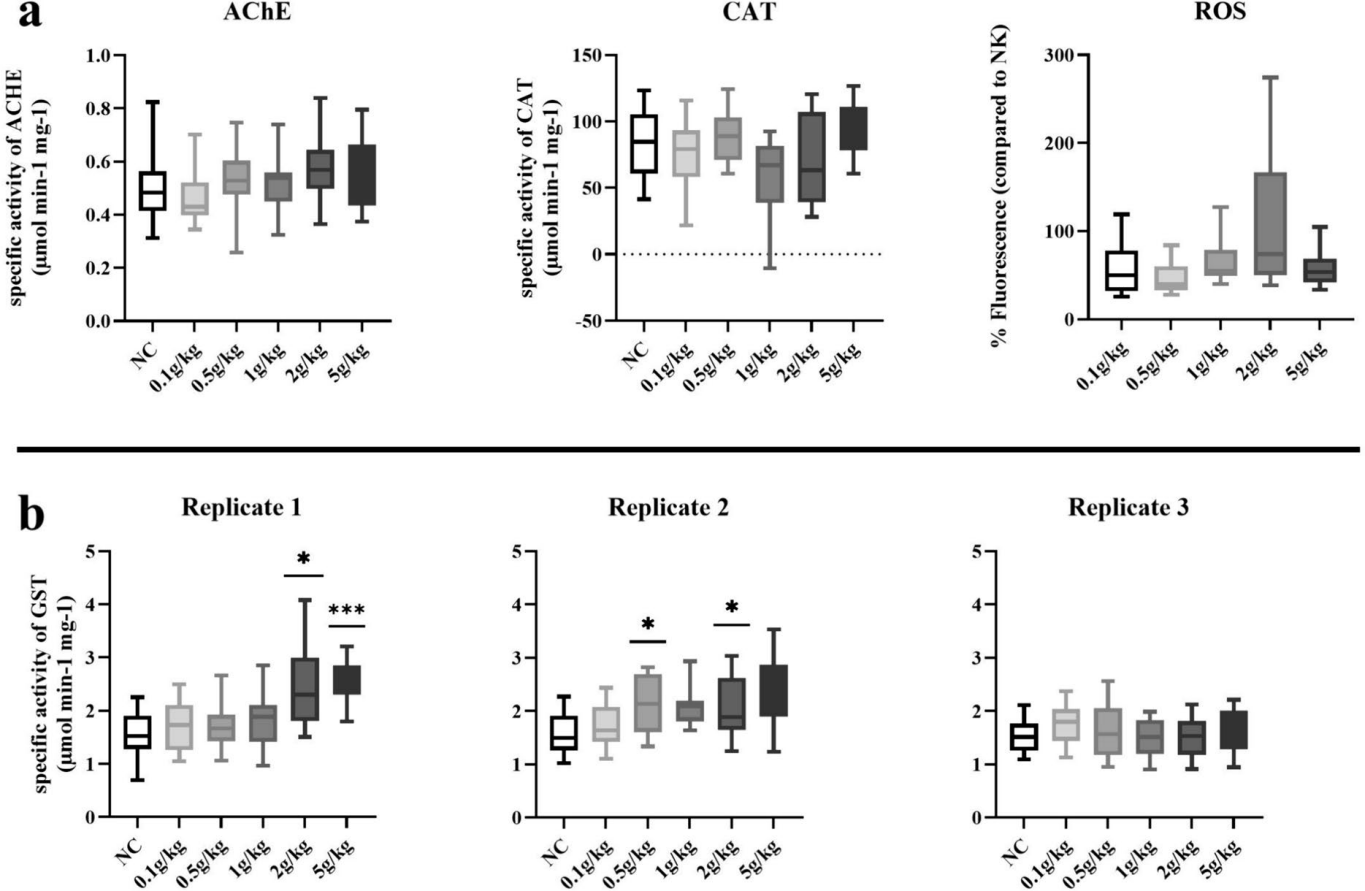
Biomarker responses after exposure to PE microplastic for 28 days. **a** Specific activity of acetylcholinesterase (AChE) and catalase (CAT) as well as relative fluorescence of reactive organic substance (ROS) measurements in *E. fetida* earthworms (mean ± standard deviation; *n* = 20). **b** Specific activity of glutathione S-transferase (GST) on three different test days. Significant differences to the control are indicated with *(*p* < 0.05), **(*p* < 0.01), and ***(*p* < 0.001)

The results of the different biomarker analyses are presented in Fig. 3. Presented are the results of different biomarker responses in earthworms after exposure to PE microplastic for 28 days representative from one test day (a) and below results from three test days for GST (b).

Replicate 3 was chosen for AChE, CAT, and ROS to be visualized in graph (a), since this test day included analyses of all concentrations and significant differences between replicates made them not suitable for pooling. Statistical results include results from all 3 to 4 test replicates.

Statistical analysis showed no significant differences in comparison to control for AChE and catalase activity as well as oxidative stress for all replicate test days. As visible in Fig. 3b for glutathione S-transferase activity, there were significant differences of the second highest (*p* = 0.0322) and highest (*p* < 0.0001) in R1 and the fourth (*p* = 0.014) and second highest concentration (*p* = 0.028) in R2, although none in the third replicate (R3). For R2, the third and highest concentration failed to reach significance by a small margin with *p* = 0.193 and *p* = 0.094, respectively.

**Fig. 4:**
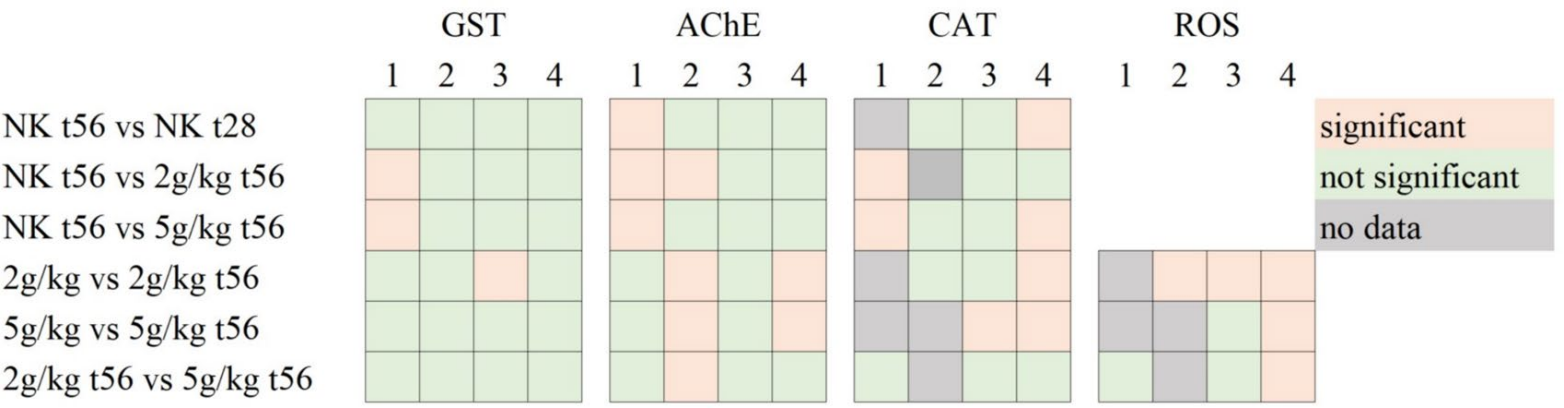
Graphical overview of the statistical testing of exposures with 2 and 5 g PE-MP/kg dry soil after 28 days of exposure (t28, not labelled) and subsequent 28 days in control soil (t56). The replicate days had an average sample size of *n* ≥ 11 with a minimum number of 5 replicates per concentration, except for R3 NK t28 with *n* = 3 in ROS analyses

In the advanced test set-up, the concentrations 5 g/kg and 2 g/kg were compared with the respective concentrations after 56 days of total test duration and their negative controls. The results did not show a consistent pattern, as visible in Fig. 4 below.

For GST activity, only 2 of 4 replicates showed induction in various comparisons. We observed a slightly increasing trend between the measured concentrations after 56 days in 2 replicates; this was however not confirmed by the other three replicates, which showed no differences to negative controls. Comparing the test days t28 and t56, no consistent trends were observed.

In the AChE measurements, we see an overall decreased activity in the negative control after 56 days for the first replicate, leading to significant differences compared to every other concentration. In the other replicates, the response from lowest to highest concentrations after 56 days shows inconsistent trends with increasing (R2) or decreasing tendencies (R4).

Catalase activity responses show significant differences in the comparison of 2 g/kg after 28 and 56 days. This was observed for two replicates, however once being higher at t28 (R4, *p* < 0.0001) and once at t56 (R3, *p* < 0.0001). The same contradictory trend was visible for 5 g/kg, significant for R4 with *p* < 0.0001.

Oxidative stress was more scarcely investigated for these samples. In the last replicate, the 28-day exposure scenario induced a higher amount of general oxidative species, however with large standard deviations also depending on the tested day. Measured fluorescence values for 56-day exposure were very low, also compared to other replicates. The second highest concentration differed in all three replicates, being consequently lower than at 28-day exposure.

## Discussion

In this study, we investigated the effects of commonly used non-degradable and biodegradable mulch films on soil fauna organisms. Therefore, several biological endpoints were chosen, such as reproduction and hatching, weight change, biomarker response, and short-term recovery. A key finding was the absence of effect on reproduction in both *F. candida* and *E. fetida*, even at highest concentrations of 10 and 5 g/kg_dw_, respectively.

### Influence on the reproduction of earthworms and springtails

Effects of polyethylene and polylactic acid/polybutylene adipate terephthalate-based mulch film microplastic on the reproductive output of two commonly used soil organism species were investigated. In both species, neither a concentration-dependent nor a polymer-dependent effect was observed. That being the case, the replicate size was assumed to play a major role in these results.

For the springtail reproduction testing, we found significantly lower amounts of produced juveniles per adult at the lowest concentration (0.1 g/kg). However, when including other conducted replicates that did not fulfill one of the validity criteria by a small deviation, there were no statistically significant differences between concentrations and negative controls. Therefore, it was concluded that the PE and PLA/PBAT mulch film microplastic did not impact the reproduction of springtail in a significant way, as observed previously by van Loon et al. (2024). In general, we can assume that only few of the total applied MPP could directly impact springtails by ingestion-derived effects, since Kim and An (2020) showed that the edible size for springtails does not exceed 66 µm.

However, as degradation proceeds, fragmentation into smaller particles enhances the amount of edible MPP, even for very slowly degrading polyethylene particles (Briassoulis et al., 2015).

Consequences can then be conveyed in changed gut microbiota or behavior (e.g., avoidance, transport) as well as offspring production and might impair ecosystem services provided by the subclass of collembola (Ju et al., 2019; Zhu et al., 2018).

In general, short-term effects up to 6 months on soil biological health are rather insignificant compared to the estimated more severe long-term effects (Brown et al., 2022). Confirming this, Huang et al. (2023) found a higher abundance of springtails after exposure to low-density PE (LDPE) and PP after 140-day exposure after no changes were observed after shorter exposure of 40 days, showing a strong tendency of MP-induced changes to increase over time. Due to the perpetual occurring accumulation of microplastic in soil, effects level concentrations, which are currently not realistic in the environment, will be approximated.

For the earthworm testing the results were more conclusive, leading to the assumption of no effect on the reproductive output in this exposure scenario of up to 5 g MPP per kg dry soil.

The current body of study of earthworm reproduction has not yet been examined with sufficient frequency to draw general conclusions and suggests that effects are often shape- and polymer-specific. Due to the size range of 50 µm to 1 mm used here, some of the particles might not be orally ingestible by the worm, since a defined size limit has not yet been established. Li et al. (2021) observed that *E. fetida* are able to ingest high-density PE (HDPE) and PP particles with a size of 400–1464 µm and 761–1660 µm, respectively. The size fraction in this study represents a mix of different occurring sizes within the range of MP particles. Similar size fractions (250 µm to 1 mm) were previously used in Rodriguez-Seijo et al. (2017), where histopathological endpoints were investigated in addition to reproduction, who found increased stress responses in the worm after exposure with 1 g/kg_dw_ of PE MPP, although no reproductive effects were present as confirmed in this study. This is in accordance with Yang et al. (2023), who found high thresholds of reproduction impediment at 20 g/kg_dw_. Accordingly, the possibility that the worms were otherwise affected cannot be ruled out here either. Our results are further in accordance with Rodriguez-Seijo et al. (2017) and Judy et al. (2019) who tested concentrations up to 10 g HDPE MP per kg_dw_ of soil and found no effect on reproduction. Kwak and An (2021) observed damage to male reproductive organs, though not female ones, at 1 g/kg_dw_ PE MPP for 21 days. To what extent this result impacts the reproductive output is not clear. However, the low size of MP being < 300 µm confirms a size-dependent toxicity due to a more likely uptake and further internal damages.

Lesser publications studied the effects of biodegradable MPP. In Forsell et al. (2024), biodegradable PBAT MPP did not affect reproduction at up to 50 g/kg_dw_, but increased growth at lower concentrations. Holzinger et al. (2023) found a significant increase in the number of cocoons and juveniles after 8 weeks’ exposure to poly-l-lactic acid at 10 and 25 g/kg_dw_. Mentioned positive effects might result from PLA and PBAT being an additional carbon source, which, as Wang et al. (2022) showed, are preferable ingested over conventional polyethylene terephthalate.

In contrast, Ding et al. (2021) projected a reduction in reproduction at 53 g/kg for PLA, exceeding the concentrations in most studies by far. Concentrations of MPP in soil in general have been detected to be as high as 67.5 g/kg (Fuller & Gautam, 2016), exceeding this threshold value.

In Holzinger et al. (2023) and Forsell et al. (2024), concentrations did not reach this high to possibly confirm these findings; however, this might also be highly dependent of plastic shape and size.

Furthermore, Huerta Lwanga et al. (2021) investigated another earthworm species, *Lumbricus terrestris*, and did not find effects on mortality, growth, or reproduction at 1 g/kg_dw_. In our study, no effect could be observed up to concentrations of 5 g/kg_dw,_ which is in accordance with before mentioned studies. It remains to be investigated, what long-term effects of biodegradable MPP on the soil fauna might be.

Lastly, we looked at the emergence of juveniles from cocoons and found no significant effects of concentration or plastic type whatsoever. Since no juveniles and only cocoons were found after exposures ended, this approach proved to be less labor-intense and time-consuming than sorting out the juveniles after an additional 4 weeks as suggested in OECD 222 (2004). This investigation also showed that most animals hatched at weeks 8 and 9 after begin of the test, which would then result in an incomplete collection of juveniles if the aim was to determine the total reproductive output. This analysis has to our knowledge not yet been conducted and may now be the basis of comparison of other studies that look further than the parental generation of earthworms.

### Influence on biomarker expression

Complementary to analyzing the reproductive output, the biomarker response of *E. fetida* to polyethylene microplastic was investigated in soil in order to target potential underlying subcellular effects. Since observed effects did not show differences between both film types, PE was chosen as the representative film due to its essentially higher occurrence in the environment. Our objective was to assess the subcellular effects of mulch film MPP, which are considered to be low due to a significant change in only one endpoint, namely GST.

Similar set-ups with *E. fetida* and PE MP exposure have been investigated by several studies with different endpoints, which found sublethal effects in terms of biomarker expression (TBARS↑) changes starting at 250 mg/kg_dw_, but no changes in catalase and only increasing GST activity at the highest concentration. In another study from this group, no biomarker expression (AChE, TBARS) below 400 mg/kg_dw_ at only 2 weeks’ exposure was found (Rodriguez-Seijo et al., 2018a and 2018b). Confirming the latter study, Wang et al. (2019) found no discernible effects on oxidative stress (CAT, SOD, POD, GST) at amendment rates ≤ 100 g/kg_dw_ after 2 weeks’ exposure.

Chen et al. (2020) detected a significant increase in CAT and AChE activity after exposure to 100–200-µm-sized LDPE in concentrations of 1g/kg_dw_, however not for 1.5g/kg_dw_. Yu et al. (2022) investigated oxidative stress elucidated by small PE and PLA microplastic particles in two comparative natural soils and found an increasing concentration-dependent trend in both soils for AChE, CAT, and GST at 5 g/kg_dw_ and rising exposure concentration up to 140 g/kg_dw_.

Interestingly, Li et al. (2021) detected a decrease in enzyme activity (SOD↓, CAT↓, GST↓) at 250 mg/kg_dw_ HDPE with different size ranges after 14 days and 28 days. Even though the activity of some enzymes recovered from 14 to 28 days, most of them were still significantly inhibited.

The existing data is not completely consistent, dealing with different somewhat exposure scenarios and investigated biomarkers as well as possible differences between density and size fraction of PE as the latter two studies showed. The presented study resembles the build-up of Rodriguez-Seijo et al. (2018a), who found an increased glutathione S-transferase activity for their highest concentrations of 1 mg/kg, whereas this study found an increase at 0.5, 2, and 5 mg/kg in replicates 1 and 2. It can therefore be concluded that an exposition to PE MPP causes a tendential increase in GST activity. This is also in concordance with Boughattas et al. (2021).

The activation of this detoxifying enzyme suggests an adaptive response to exposure to microplastic particles, which might exert oxidative damage. Further, we hypothesize that measured stress might not only result from external physical impairment but also uptake. MPP was recovered from defecation in most exposure scenarios, when worms were depurated prior to biomarker analyses. The mulch film MPP were unaged and unprocessed, making exposure to film-adsorbed additives or other chemicals on the surface possible.

In the study from Rodriguez-Seijo et al. (2018a), catalase was also not found to be atypically active. Acetylcholine esterase was activated when small MPP were analyzed, but our highest concentration might just be around the NOEC value when looking at Yu et al. (2022). However, the main factor here does seem to be the particle size, which leads to higher impact at lower levels as investigated by the mentioned studies. This would only be logical, considering their easier translocation and uptake into organisms and even cells. Size-dependent toxicity was also confirmed for *E. fetida* by previous studies like Xiao et al. (2022).

No consistent significant differences were found for biomarker measurement after 28 days of exposure and subsequently 28 days of recovered earthworms. However, since the effect strength of biomarkers was very low after 28-day exposure, the differences were not expected to be significant, when recovery was intended to regulate to normal stress levels. There are no studies comparable to our experiment with MPP. Studies like Feng et al. (2015), who investigated recovery from pesticide exposure with this method, found that the 4 weeks’ recovery was not sufficient to reach stress levels before the exposure period. Weight changes (not presented) during exposure indicate a smaller weight gain after 28-day initial exposure, which might also be an artifact from a too short time of investigation and total recovery. Thus, our third objective results in no observation of recovery from exposure and can be a basis for more long-term investigation of environmental resilience against MPP pollution.

### Future perspectives

Differences between conventional PE and biodegradable PLA/PBAT mulch film MPP were not observed in this study. This is due to the lack of effects in exposure, which itself results in a similar assessment for this scenario. The broader context in which this study was conducted came to a similar conclusion (Wolf et al., unpublished). Even though this study did not cover effect thresholds that were, e.g., predicted by Ding et al. (2021), accumulation of (micro)plastic particles in the environment will likely pose a threat in the future due to the lack of removal possibility, thus pushing the idea of environmentally degradable substances.

However, several studies from the last years have shown incomplete degradation under realistic field conditions (Wolf et al. (unpublished), Li et al. (2023), Sintim et al. (2020)). In cool climates, it could take many years to degrade fragments of BDM. Even now, in Tunisian agricultural soils with mulching, PBAT was the second most commonly detected polymer type (Boughattas et al., 2021).

One of the more easily adaptable influencing factors of microplastic creation is the film thickness as demonstrated by Steinmetz et al. (2022) and Xiong et al. (2023). For traditional films, it is thus advisable to establish a minimum thickness limit for the material to minimize potential losses during film collection. Another approach to consider is a substantial increase in the thickness of biodegradable films and their subsequent retrieval from the field post-use. The remaining particles on the field are then possibly degraded over time in contrast to their non-degradable counterpart. BDM utilization would then shift to repeated instead of one-time application, which would have several other implications to discuss. The degradation of applied mulch films should be discussed in conclusion and not just applied to artificial set-up regimes with high bacteria availability and environmentally unrealistic temperatures as it is done for the compostability labelling. This is especially relevant for the investigated scenario, since mulch films are in direct contact with nature but also human food.

## Conclusion

In this study, we found no effects of microplastic particles derived from biobased and degradable as well as conventional mulch film on earthworm and collembolan species. Earthworm reproduction was not impacted, but increasing glutathione S-transferase activities with rising PE microplastic amount indicate a higher necessity for detoxification within the organism. Further studies with PLA/PBAT could be interesting to give insight into polymer-derived biomarker activity differences. Collembola were not affected by any investigated exposure scenario.

Overall, the applied tests show that mulch film–derived microplastic of both origins does not represent a threat in this single stressor scenario. The used concentrations exceed the currently known hot spot concentrations of microplastic on agricultural land by far, leading to the conclusion of no acute risk for this scenario. However, many factors could not be targeted in this laboratory study, such as naturally occurring multiple stressors, species interaction, and fate and behavior of particles. Frequent change in weather conditions such as rain events and temperature regime change might influence the toxicity in terms of leachable substances and degradation impairment. When further investigated, interdisciplinary research should also involve the interaction of agricultural microplastic with co-exposed substances such as pesticides that might alter the effects of both contaminants onto non-target organisms on the field.

## Electronic supplementary material

Below is the link to the electronic supplementary material.

Supplementary file1 (PDF 173 KB)

Supplementary file2 (XLSX 9 KB)

Supplementary file3 (XLSX 9 KB)

Supplementary file4 (XLSX 9 KB)

## Figures and Tables

**Fig. 5:**
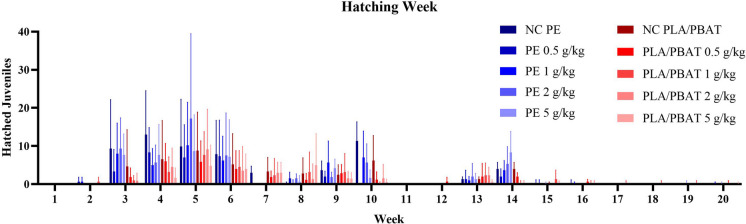
Hatching week of juvenile from cocoons after 28 d exposure to different concentrations of polyethylene and polylactic acid/polybutylene adipate terephthalate microplastic. There were no differences in hatching behavior observed

## Data Availability

The data that support the findings of this study are available from the corresponding author upon reasonable request.

## Appendix

See Tables 2, 3 and 4

**Table 2:** Overview of the soil properties from different deliveries of the RefeSl 01-A from Fraunhofer Institute for Molecular Biology and Applied Ecology IME (Schmallenberg, GER). *according to DIN ISO 11277

Physiochemical properties	RefeSol 01-A (03.02.2020)	RefeSol 01-A (05.05.2020)	RefeSol 01-A (22.05.2020)	RefeSol 01-A (31.05.2020)	RefeSol 01-A (11.06.2021)	RefeSol 01-A (13.09.2021)	Mean ± SD
Sand (%)*	73.1	69.8	73.1	69.8	69.8	69.8	70.9 ± 1.6
Silt (%)*	19.8	24.4	19.8	24.4	24.4	24.4	22.8 ± 2.2
Clay (%)*	7.0	5.9	7.0	5.9	5.9	5.9	6.3 ± 0.5
C_org_ (%)	0.96	0.95	0.96	0.95	0.95	0.95	0.95 ± 0.004
Total N (g/kg)	0.9	0.81	0.9	0.81	0.81	0.81	0.84 ± 0.04
pH_CaCl2_	5.65	5.72	5.65	5.72	5.72	5.72	5.69 ± 0.03
KAK_eff_ (mmol/kg)	3.5	40.0	7.0	40	40	40	28.4 ± 16.4
Water holding capacity (g/kg)	293	287	293	287	287	287	289 ± 2.8
Biomass (mg/kg C_mik_)	198	174	198	174	174	174	182 ± 11.3

**Table 3:** Size distribution of the polyethylene microplastic used. Feret,min describes the minimum size of the MPP in μm. Feret,max describes the maximum size of the MPP in μm. Only particles to a size range of 1000 µm were used in testing

Feret,min [μm]	Number Particles (Feret, min)	Feret,max [μm]	Number Particles (Feret, max)
50–100	3	150–200	2
100–150	7	200–400	12
150–200	4	400–600	15
200–400	26	600–1000	28
400–600	19	1000–1500	28
600–1000	30	1500–2000	13
1000–1500	29	2000–3000	28
1500–2000	13	3000–4000	7
2000–3000	2		

**Table 4:** Size distribution of the polylactic acid/polybutylene adipate terephthalate microplastic used. Feret,min describes the minimum size of the MPP in μm. Feret,max describes the maximum size of the MPP in μm

Feret,min [μm]	Number Particles (Feret, min)	Feret,max [μm]	Number Particles (Feret, max)
5–15	1	50–100	8
15–25	2	100–150	5
25–50	5	150–200	12
50–100	22	200–400	59
100–150	20	400–600	66
150–200	28	600–1000	61
200–400	96	1000–1500	58
400–600	42	1500–2000	21
600–1000	65	2000–3000	20
1000–1500	14		
1500–2000	14		
2000–3000	1		

See Fig. 5

## Abbreviations

AChE: Acetylcholine esterase
ANOVA: Analysis of variance
BDM: Biodegradable mulch film
CAT: Catalase
DMSO: Dimethylsulfoxide
GST: Glutathione S-transferase
MP: Microplastic
MPP: Microplastic particles
PBAT: Polybutylene adipate terephthalate
(HD/LD) PE: (High/low density) polyethylene
PLA: Polylactic acid
PLLA: Poly-l-lactic acid
POD: Peroxidase
PP: Polypropylene
ROS: Reactive oxygen species
SOD: Superoxide dismutase
TBARS: Thiobarbituric acid reactive substances
