# Supplementary figures and images for: Different mulch films, consistent results: soil fauna responses to microplastic
Source: Environ Monit Assess. 2024 Sep 18;196(10):943. doi: 10.1007/s10661-024-13096-x (PMC11408579; doi:10.1007/s10661-024-13096-x)

## Hatching Week

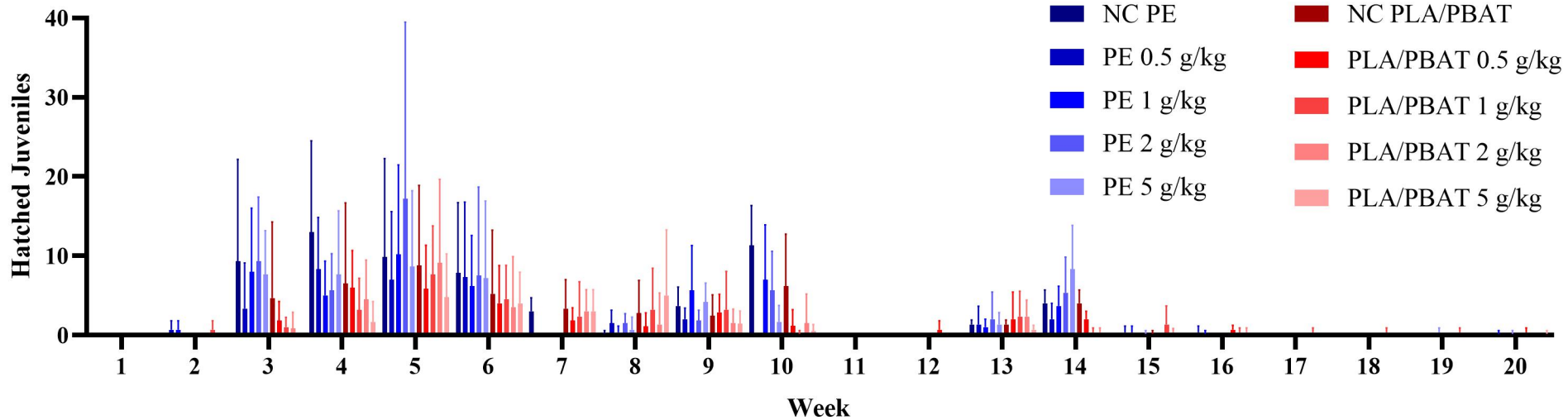

Supplement: Supplementary file 1 — Supplementary file1 (PDF 173 KB) [file 10661_2024_13096_MOESM1_ESM.pdf]
